# Skin Autofluorescence Based Decision Tree in Detection of Impaired Glucose Tolerance and Diabetes

**DOI:** 10.1371/journal.pone.0065592

**Published:** 2013-06-04

**Authors:** Andries J. Smit, Jitske M. Smit, Gijs J. Botterblom, Douwe J. Mulder

**Affiliations:** 1 Department of Medicine, University Medical Center Groningen, and University of Groningen, Groningen, The Netherlands; 2 Department of Medicine, Gelre Ziekenhuis, Apeldoorn, The Netherlands; INRCA, Italy

## Abstract

**Aim:**

Diabetes (DM) and impaired glucose tolerance (IGT) detection are conventionally based on glycemic criteria. Skin autofluorescence (SAF) is a noninvasive proxy of tissue accumulation of advanced glycation endproducts (AGE) which are considered to be a carrier of glycometabolic memory. We compared SAF and a SAF-based decision tree (SAF-DM) with fasting plasma glucose (FPG) and HbA1c, and additionally with the Finnish Diabetes Risk Score (FINDRISC) questionnaire±FPG for detection of oral glucose tolerance test (OGTT)- or HbA1c-defined IGT and diabetes in intermediate risk persons.

**Methods:**

Participants had ≥1 metabolic syndrome criteria. They underwent an OGTT, HbA1c, SAF and FINDRISC, in adition to SAF-DM which includes SAF, age, BMI, and conditional questions on DM family history, antihypertensives, renal or cardiovascular disease events (CVE).

**Results:**

218 persons, age 56 yr, 128M/90F, 97 with previous CVE, participated. With OGTT 28 had DM, 46 IGT, 41 impaired fasting glucose, 103 normal glucose tolerance. SAF alone revealed 23 false positives (FP), 34 false negatives (FN) (sensitivity (S) 68%; specificity (SP) 86%). With SAF-DM, FP were reduced to 18, FN to 16 (5 with DM) (S 82%; SP 89%). HbA1c scored 48 FP, 18 FN (S 80%; SP 75%). Using HbA1c-defined DM-IGT/suspicion ≥6%/42 mmol/mol, SAF-DM scored 33 FP, 24 FN (4 DM) (S76%; SP72%), FPG 29 FP, 41 FN (S71%; SP80%). FINDRISC≥10 points as detection of HbA1c-based diabetes/suspicion scored 79 FP, 23 FN (S 69%; SP 45%).

**Conclusion:**

SAF-DM is superior to FPG and non-inferior to HbA1c to detect diabetes/IGT in intermediate-risk persons. SAF-DM’s value for diabetes/IGT screening is further supported by its established performance in predicting diabetic complications.

## Introduction

Despite the major, woldwide increase in type 2 diabetes mellitus (T2DM), and its preceding stages impaired fasting glucose (IFG) and glucose tolerance, almost half of those affected are not aware of having this condition (Eckardstein) [1]–[3]. This leaves a long clinically latent period in which T2DM and IGT can be detected, for diabetes alone this is estimated to be 5 years. Diagnosis of T2DM is still based on glycemic criteria, in the WHO criteria and Europe on glucose levels. An International Expert Committee (IEC) recently proposed new diagnostic criteria based on measurement of A1C, with A1C≥6.5%/48 mmol/mol for diabetes and 6.0–6.4%/42–46 mmol/mol for “high risk” of progression to diabetes [4], [5]. The American Diabetes Association (ADA) subsequently proposed HbA1c≥6.5% for the diagnosis of diabetes and 5.7–6.4%/39–46 mmol/mol for the highest risk to progress to diabetes.

Early detection and screening for IGT and DM are warranted because timely start of treatment in IGT may retard further development to DM, and once DM is present may prevent complications [6], [7]. Many T2DM screening procedures and scores have been developed, but several difficulties and limitations make their broader use challenging: this includes matters like limited response rates, limited availability and invasiveness of required (confirmatory) tests (e.g. OGTT) which are not routinely available and which also suffer from limited reproducibility [1]. In Europe, the use of questionnaires like the FINDRISC or Cambridge Diabetes Risk score (CDRS) has been proposed as first step in diabetes screening, followed by a plasma glucose step, fasting or preferably with an OGTT, using WHO defined cut-off values, as confirmatory test [8], [9]. The importance, given in recent European guidelines, to early interventions to prevent development of diabetes also stresses the need for alternative technologies for easy detection of IGT and DM [10], [11].

Previously we showed in skin biopsy validation studies that noninvasive SAF is a proxy for tissue accumulation of advanced glycation endproducts (AGE) [12], [13]. Holman et al proposed AGE to be the prime candidate as carrier for the so-called metabolic legacy effect [14], [15]. This carrier effect of AGE has been invoked as explanation for the prolonged and late beneficial effects of an early, temporary period of intensified glycemic control: accumulation of AGE in tissues with slow turnover, persisting there for many years, may be prevented or retarded by early improved glycemic control [14]. In the preceding stages of diabetes, intermittent periods of (post-meal) hyperglycemia in impaired glucose tolerance might similarly result in persistent increases in tissue AGE and skin AF levels. Thus, skin AF is a candidate tool to detect IGT and diabetes in periods of (intermittent) hyperglycemia. A study by Maynard et al in naive persons indeed reported that noninvasive skin AF performed better than glycemic criteria (FPG and HbA1c) in detecting OGTT-confirmed diabetes [14]. However, some caveats are warranted concerning the specificity of skin AF: AGE may also be rapidly formed during oxidative stress, and elevated SAF levels have been reported after cardiovascular events, serious infections, and in autoimmune disease [17], [18]. In renal failure, diminished renal excretion of AGE free adducts and AGE peptides is another cause of increased plasma and tissue AGE levels and skin AF [19]. Accounting such alternative reasons for elevated skin AF might improve specificity for diabetes and IGT detection especially in intermediate risk persons with comorbidity. On the other hand, decision trees (including age and systolic bloodpressure) have also been proposed as simple and reliable tools for identifying individuals with IGT or T2DM [20], while other well-known risk factors for IGT/DM such as BMI, and family history of diabetes are represented in most of the diabetes screening questionnaires [8], [9]. We assumed that the performance of noninvasive skin AF-based DM/IGT decision tree might also be improved by integrating such easily available items.

Therefore, we aimed to develop and validate a skin AF based decision tree to detect IGT or diabetes in a group of subjects at intermediate risk. Test characteristics were compared to those of conventional diabetes diagnostic tools including FPG and HbA1c. In addition, we also compared it to the FINDRISC questionnaire alone or in combination with FPG.

## Methods

### Ethics Statement

The study protocol was approved by the IRB (METc) of the University Medical Center Groningen (METc c2009-367), and placed in the clinical trials.gov register NCT01406665. Informed written study consent was obtained from all participants, and the study was conducted according to the principles expressed in the Declaration of Helsinki.

### AGE Reader and Skin Autofluorescence

The AGE Reader (DiagnOptics Technologies BV, Groningen, The Netherlands) is based on the non-invasive measurement of skin autofluorescence (AF). Skin AF has been compared with specific AGE in the dermis of skin biopsies from the measurement site in patients ith diabetes, renal failure or healthypersons from a wide age range. In a combined analysis of these three studies 76% of variance in skin AF level could be explained by the variance of dermal skin biopsy pentosidine levels. Lower, but still highly significant relations were also found with CML (a non-fluorescent AGE) and CEL [10]. Measurements are performed at the volar site of the forearm where the illuminating light enters the skin almost perpendicularly over an area of ∼4 cm^2^. This excitation light is in the wavelength range of 350–420 nm (maximum intensity approximately at 370 nm). The AGE Reader uses a spectrometer manufactured by Avantes BV (Eerbeek, The Netherlands) to measure the light that is reflected and emitted from the skin. Dark and white reference measurements take place during each measurement to correct for detector properties and background light, and to calculate skin reflectance. The device has a completely automated measuring procedure of <1 min, results are presented immediately afterwards. The skin used for the measurement has to be visibly normal, and not too dark of colour (see also exclusion criteria), because irregularities and pigmentation can influence the measurement. Reliable skin AF measurements can be performed in persons with reflectance values >6%, this covers Fitzpatrick skin color classes I-V. The AGE reader automatically reports unreliable measurements in case of lower reflectance levels; the handling of this result in evaluation of the decision tree is described below. In persons with reflectance levels between >6–10%, AGE reader software version 2.3 was used for skin color independent assessment of skin AF as decribed by Koetsier [22]. AF is expressed in Arbitrary Units (AU). AF was measured using the triple measurements setting with 3 measurements of approximately 20 seconds on 3 different lower arm sites at one of the arms, and the mean value of these 3 AF measurements was used for analysis. Reproducibility was earlier tested by Meerwaldt et al in 25 healthy persons and 25 diabetic patients, which showed a mean relative error in AF of 5.8% on a single day [13].

### Development of Decision Model

#### Use of skin AF age percentile cut-off levels alone

Age percentiles are based on the reference value data by Koetsier et al [17]. Skin AF increases linearly with age, according to skin AF = 0.024× age +0.83. This formula has been based on a study in 428 persons with normal renal function and glucose values, without any known health problems, and without abnormalities during screening physical exam. For persons >50 years, the choice of cut-off level ≥70^th^ age percentile for skin AF was based on data distributions of skin AF levels previously reported by Lutgers et al for persons with and without established diabetes [23]. For persons ≤50 years the choice of a higher cut-off level ≥80^th^ age percentile was not based on analysis of previously reported data distributions, but on the lower risk of diabetes in this age group.

#### Decison model for detection of IGT/diabetes, based on skin AF age percentile, BMI, and conditionally questions

**Figure 1:**
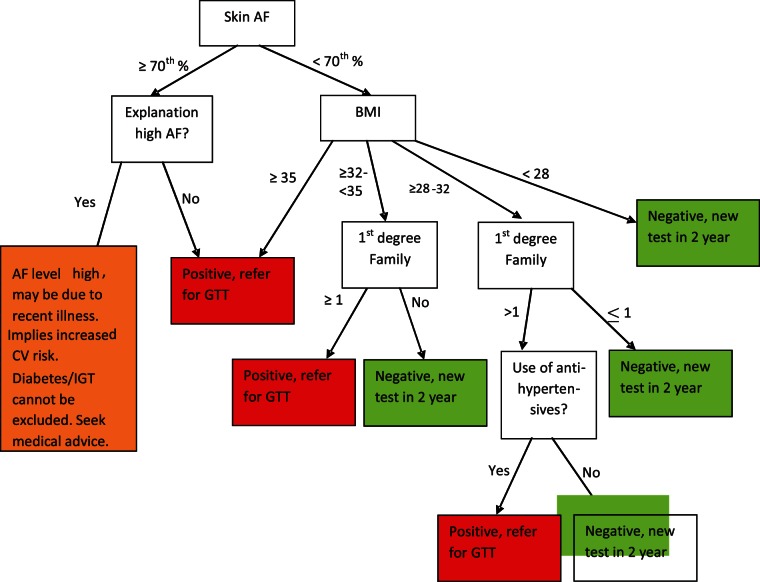
Summary design of skin AF based IGT/diabetes detection decision model (SAF-DM), using SAF level age-dependent cutoff levels (shown here for age >50 years, different cut-off levels at lower age), using age percentiles. Effects of low reflectance have not been included.

The summary design of this model is shown in Figure 1. The model adds, depending on the measured skin AF age percentile, question-based information which may be obtained readily in a screening setting. In case of elevated skin AF age percentile, maximally three questions are asked about conditions possibly leading to increased skin AF levels: questions ask for a recent history of serious (leading to hospital admission) infections (<1 year) or CVE (<2 years), or a history of renal or autoimmune disease. In case of documented and ongoing peripheral arterial or coronary disease (stable angina pectoris) a recent admission was not required. Further, in case skin AF is below age percentile cut-off levels questions are asked on classical IGT/diabetes risk factors apart from age: questions on weight/BMI, on 1^st^ degree family history of diabetes (and if so, the number), and on use of antihypertensives. These questions were formulated and asked for in the same manner as in the FINDRISC questionnaire [8]. Scoring in the developed decision model first depends on a >70^th^ (<50 years) or >80^th^ (≥50 years) age percentile cut-off level of skin AF, plus information on the questions described above, and on BMI with different cut-off levels: at >35 kg/m2, >32 kg/m2, and >29 kg/m2, respectively. Persons with a BMI>35 kg/m2 were assumed to have diabetes/IGT, independent of other information. For the latter two BMI groups, a combined weighing of BMI, skin AF percentile and information on number of first degree relatives with diabetes and on antihypertensive use determined whether the decision tree outcome gave a positive score for IGT/diabetes. This combined risk weighing was based on the risk assumptions for the three variables BMI, family history and skin AF derived from data of questionnaire-based risk estimates (FINDRISC and Cambridge Diabetes Risk Score, CDRS). In the persons with an elevated skin AF above the defined age percentile cutoff, the then asked questions described above, are accounted for as follows. If one of the questions on an alternative condition in the last year was positively answered, the decision tree result states that diabetes/IGT may be present, but alternative explanations for the abnormal results are available. In the decision tree performance analysis below such a response was scored as negative for IGT/diabetes.

Finally, spectral information measured by the AGE reader in addition to the skin AF is also used, especially skin reflectance levels. As described above, if skin reflectance was ≤6%, and, thus, the measurement rejected, the decision tree result states that diabetes/IGT may be present, but alternative methods for assessing diabetes-IGT risk are needed. Such a response was scored as negative for IGT/diabetes in the Results.

### Patients

Participants fulfilled the following inclusion criteria: males and females aged 20–80 years with an a priori intermediate risk for IGT/diabetes defined by: presence of at least one criterion from the NCEP definitions for metabolic syndrome (except obviously high FPG) [24]; or at least once having had an increased glucose or glycated hemoglobin value in the preceding 2 years, but these below the range of diabetes/IGT. Exclusion criteria were known diabetes mellitus, use of oral antidiabetics (for other purposes than diabetes such as non-alcoholic fatty liver disease), local skin disease of the lower arm obviating SAF measurement, known serious renal insufficiency (s-creatinine >150 umol/l).

Participants were recruited from vascular and general internal medicine outpatient clinics of the Gelre hospital Apeldoorn (n = 101) and the University Medical Center Groningen (n = 117).

### Study Procedures: Oral Glucose Tolerance Test (OGTT), SAF, HbA1c, and FINDRISC

On the days preceding participation no important changes in food habits were allowed, especially no carbohydrate restriction. From the early evening before testing no alcohol or heavy meals were allowed, only light snacks and low caloric drinks. The participants came in a fasting state (at least 8 hours) in the morning. Blood was drawn 15 min after arrival from an antebrachial vein for venous plasma glucose and glycated hemoglobin, and serum creatinine assessment. Then, the skin AF measurement was performed. Thereafter the glucose load of 75 g of glucose solved in 150 ml water was given to drink. If wished by the participants, they were allowed to drink or wash their mouth with another 100 ml of water. No meals or drinks were allowed in the subsequent two hours, at least until the final blood samples had been drawn. Two hours after the oral glucose administration another glucose sample was taken and a second skin AF measurement performed. Participants avoided physical exercise during this period.

Before or during the test body length, weight, waist circumference, blood pressure, heart rate, family history for diabetes and for cardiovascular disease, and smoking status were collected. All participants filled in the Finnish version of the FINDRISC questionnaire. A score ≥10 points was considered to signify diabetes, or suspicion thereof, and was used in the analysis below [8]; an alternative approach used a cutoff level ≥7 points, followed by a FPG.

Routine laboratory procedures were used for the assay of venous plasma glucose, serum creatinine and glycated haemoglobin.

Levels of glucose tolerance were defined according to WHO-GTT, or to 2009 IEC criteria: the IGT group according to WHO-GTT fulfilled the t = 120 min criterium of plasma glucose ≥7,8–11.0 mmol/L, with either a FPG level above or below 5.6 mmol/l. For IEC criteria, an HbA1c≥6.0%/42 mmol/mol defined suspicion of diabetes [4]. Diabetes definitions were in line with reported WHO-GTT, or the 2009 IEC criteria, respectively.

For assessing test characteristics of the methods compared (FPG, HbA1c, skin AF alone and skin AF-based decision tree, FINDRISC) the categories diabetes, and IGT or suspicion, were combined. For the HbA1c the WHO-GTT criteria were used, for performance of FPG, the 2009 IEC criteria, using HbA1c levels were used in comparison [4].

### Sample Size and Statistical Analysis

#### Sample size

Using the in- and exclusion criteria above for an intermediate risk group in an expected mainly middle aged to elderly group, it was estimated that the risk for diabetes/IGT, as defined by an OGTT, would be 25–30%. In such an intermediate risk group, misclassification was assumed to be 35% for cut-off levels of fasting plasma glucose >6 mmol/l (in a previously analysed pilot high risk sample it was 30%,unpublished observations), and 35% for HbA1c≥6%/42 mmol/mol [24]. Assuming this 35% misclassification rate for HbA1c in this setting, and 22% for the decision tree, a sample size of 148 persons would be needed to establish this with a p<0.05 and a power of 80%. After estimated correction for drop-out, we aimed to include 180 persons.

#### Statistical and performance analysis

Mean and SD are given in case of normal distribution, otherwise median and range.

The screening performance of FPG and HbA1C tests, of the FINDRISC score ≥10 points, and of the skin AF alone and the skin AF based decision tree, were assessed by comparing their respective sensitivities and specificity to detect diabetes or IGT/suspicion.

## Results

218 persons with an intermediate risk of having diabetes or impaired glucose tolerance were included in the study. Characteristics of this group are given in Table 1. Mean age was 55.9±10,4 yr (33<45 yr, 23>70 yr). With OGTT-based WHO criteria 28 had diabetes, 46 IGT, 41 IFG, 103 normal glucose tolerance. Of the patients with diabetes, 2 had an HbA1c<6%/42 mmol/mol, and 19 an HbA1c level between 6.0–<6.5%/42–48 mmol/mol. Of the 46 persons with IGT, 16 had an HbA1c<6%/42 mmol/mol. With HbA1c-based International Expert Committee-2009 (IEC-2009) criteria, 13 had diabetes, 87 suspicon, and 118 normal glucose tolerance. 6 of these 13 diabetes patients had normal glucose tolerance according to the WHO-OGTT criteria.

**Table 1:** Patient characteristics.

Age (years)	55,9 (10,4)
Male	129/218 (59%)
DM/IGT according to OGTT (WHO criteria)	28 (13%)/46 (21%)
DM/suspicion according to HbA1c (IEC 2009 criteria)	13 (6%)/87 (40%)
Glucose (mmol/l)	5,8±0,8/41±15
HbA1c (%/mmol/mol)	5,9±0,4
Smoking (N, yes/former/current)	66/75/77
BMI (kg/m2)	28,4±4,9
Waist circumerence (cm)	101±12,1
s-creatinine (umol/l)	82±18 (40–166)
Previous CV events	97 (44%)
Family 1^st^ degree diabetes (n;%)	82 (38%)
Total cholesterol	4,67±2
HDL cholesterol	1,2±0,36
LDL cholesterol	2,8±1,5
Systolic BP (mmHg)	140±20 (80–227)
Diastolic BP (mmHg)	80±12 (40–107)
Antihypertensive medication	169 (78%)

Values are mean ± S.D (plus range or number) or percentage; AF: Autofluorescence; DM: diabetes mellitus; Former smoker: smoked in the past 10 years, last year excluded; BP: blood pressure; BMI: Body Mass Index.

82 persons had a 1-st degree family member with DM (no data on distribution type 1 vs type 2), 23 of them had >1 1-st degree family members with DM. In 75 of the 97 persons with a CV event this had occurred <2 years previously or was still active. 29 persons had a history of kidney disease, 5 of them were known to have CKD ≥ class III. 2 persons identified themselves to have an autoimmune disease.

27 persons had a FINDRISC score <7 points, 61 had a score of 7–9 points, 130 had a score ≥10 points.

Mean AF was 2.41±0.62 (range 1–4.87 AU), the study participants were at a mean 61^st^ percentile for their age. Mean reflectance was at 16±6 percent (range 3–43%; 4≤6%).

Using AF alone to detect OGTT-based diabetes/IGT, 57 were misclassified, with 23 false positives (FP), 34 false negatives (FN), and a sensitivity (S) of 68% and specificity (SP) of 86%. Using AF alone to detect IEC-2009 HbA1c-based diabetes/suspicion, 93 were misclassified, with 43 FP, 50 FN, with an S of 50% and a SP of 64%. Using SAF-DM to detect OGTT-based diabetes/IGT, FP was reduced to 18, FN to 16 (of which 5 with DM), with a sensitivity (S) of 82% and specificity (SP) of 89%. Using SAF-DM to detect IEC-2009 HbA1c-based diabetes/suspicion, FP was reduced to 33, FN to 24 (4 for DM), resulting in a S of 76% and a SP of 72%. For FPG, using IEC-criteria for DM/suspicion, 70 were misclassified (29 FP, 41 FN; S 59%, SP 75%). If information on BMI and family history would have been added in a similar way as in the SAF-DM decision tree, the number of FN would have fallen with 5, but that of FP would have risen with 14 to 43, with a resulting S of 80% and SP of 64% For HbA1c using OGTT criteria, 66 were misclassified (48 FP, 18 FN; S 80%, SP 75%). Again, if information on BMI and family history would have been added in a similar way as in the SAF-DM decision tree, the number of FN would have fallen with 3 (1 DM, 2 IGT), that of FP would have risen with 24, with a resulting S of 80% and SP of 50%.

As for the FINDRISC score ≥10 points, 102 persons were misclassified for detection of OGTT-based diabetes/IGT, with 79 FP and 23 FN, S 69% and SP 45%. With a FINDRISC score ≥10 points, using IEC-criteria for DM/suspicion, 119 were misclassified (90 FP, 29 FN; S 71%, SP 24%). If, following a two-step approach, a FINDRISC score ≥7 would qualify for a subsequent FPG, for detection of OGTT-based diabetes/IGT, 77 persons would be misclassified (32 FN, 45 FP) (S 57%; SP 69%). If, following a two-step approach, a FINDRISC score ≥7 would qualify for a subsequent FPG, for detection of IEC-based diabetes/suspicion, 70 persons would be misclassified (48 FN, 22 FP) (S 52%; SP 81%).

**Figure 2:**
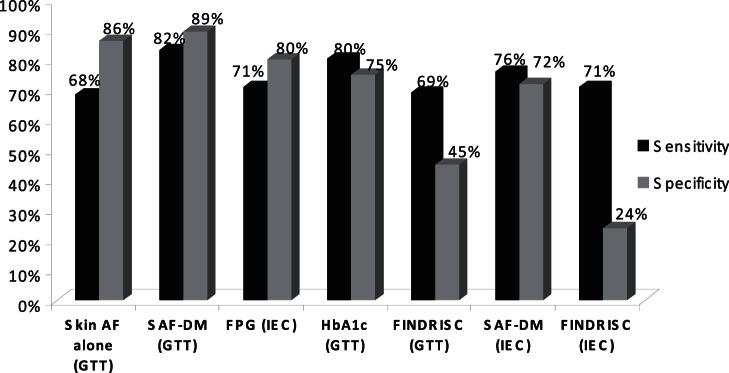
Sensitivity and specificity of skin AF alone, skin AF based decision tree (SAF-DM), FPG, HbA1c and FINDRISC for correct classification of diabetes/IGT versus normal, using OGTT based WHO criteria, or for DM/suspicion DM using HbA1c based IEC 2009 criteria.

Part of these results are also shown, for the decision tree (SAF-DM) in comparison with FPG, HbA1c and FINDRISC, in table 2 and figures 2.

**Table 2:** Diagnostic performance of skin autofluorescence alone (SAF), the skin autofluorescence based decision-tree (SAF-DM), FPG, HbA1c and FINDRISC score ≥10 points, compared to WHO-GTT defined diabetes-IGT, or IEC 2009 HbA1c-defined diabetes/suspicion.

	SAF vs OGTT	SAF-DM vs OGTT	SAF vs IEC/HbA1c	SAF-DM vs IEC HbA1c	FPG vs IEC/HbA1c	HbA1c vs OGTT	Find-risk ≥10 vs OGTT	Find-risk ≥10 vs IEC/HbA1c
FP (n)	23	18	43	33	29	48	79	90
FN (n)	34	16	50	24	41	18	23	29
S (%)	68	82	50	76	71	80	69	71
SP (%)	86	89	64	72	80	75	45	24

The number of false positives (FP;n) and false negatives (FN;n), an sensitivity (S) and specificity (SP) are shown.

## Discussion

The current study shows that in persons with an intermediate risk for IGT or diabetes mellitus, a skin AF-based decision model performs better than the conventional diabetes detection tools FPG, and than the FINDRISC questionnaire alone, or their two-step combination, and equally to HbA1c in detecting IGT or diabetes.

Previous results on the use of skin AF in detecting diabetes, reported by Maynard et al, collected in naive persons, are now confirmed [16]. Risk prediction is even more accurate in our higher risk group by using the SAF-DM decision tree. Several differences exist between the studies, not only in our use of an intermediate risk group instead of a naive group. The initial choice for the intermediate risk group reflects the policy in many countries to approach early diabetes detection not by population screening but by selective or targeted screening in groups with increased risk, or even opportunistic screening or case finding [25]. Age, and characteristics related to overweight or metabolic syndrome, are usual criteria for such a selected screening. We chose to use the presence of at least one metabolic syndrome criterium, or a historical non-diabetic elevated glucose value as entry criteria. These criteria are also used in some of the questionnaires which are used as initial step in diabetes screening programs. We agree that these study entry selection criteria form one of the limitations of our study, and a follow-up study in lower risk groups and in the general population should be performed. Some of such studies are currently ongoing. However, our approach resembles the selected screening approach currently used in many countries.

A second major difference with the study by Maynard et al is that in the present study skin AF was considered in conjunction with some other known and easily retrievable risk factors for diabetes, and resulted in an improvement in diagnostic performance. In the Maynard study the measured skin AF is also corrected for calendar age, but the procedure for this age correction is not made explicit. We used a previously defined 70% or 80% age percentile cut-off value. Additionally and probably more important, we incorporated other readily available information on well-known other diabetes risk predictors such as BMI, and conditionally family history of diabetes, and use of antihypertensives in our skin AF based decision tree. These predictors have been shown to have additional predictive value, and are also part of initial diabetes screening tools like the conventional diabetes risk questionnaires (such as FINDRISC or Diabetes Risk Score) [8], [9]. The improvement in diagnostic performance is also confirmed in our study in the comparison with use of the age-corrected skin AF alone. The size of our group was not large enough to reliably perform additional modeling on the chosen cutoff levels for BMI subclasses, additional lower skin AF age percentile subcategories, and answers to separate questions on comorbidity. Future data from ongoing studies may allow to do so. While obesity is unequivocally related to diabetes risk, in earlier studies skin AF in persons both with and without diabetes was only modestly related to BMI [23].

A third difference deals with alternative causes for high skin AF levels. Elevated skin AF is found not only in diabetes or IGT, but also in several other conditions such as infections, acute cardiovascular events and autoimmune disease occurring in the period (of at least months) preceding the skin AF measurement [17], [18]. In our decision tree we used additional questions in those with an elevated skin AF (>70^th^ or 80^th^ percentile) level to detect possible other factors than glycemic stress leading to increased skin AF and AGE levels. Obviously, the aim is to diminish the number of persons with a false positive result on screening for diabetes/IGT defined by glycemic criteria. However, such an elevated skin AF nevertheless has been shown to be a strong marker of increased cardiovascular risk in several of these alternative conditions such as renal failure, autoimmune disease, and previous cardiovascular disease. Thus, the elevated skin AF may still be considered to be valuable as a tool for CV risk prediction even when no diabetes/IGT is present.

Our study confirms the well-known lack of concordance between the conventional glycemic criteria [25], [26]. A substantial part of persons identified with one of the glycemic tests to have diabetes or IGT scores normal glucose tolerance with the other criteria. We obviously used the WHO-GTT criteria to test the performance of HbA1c, while IEC criteria were used to score the performance of FPG, since the FPG partly defines the criteria of the ‘gold standard’ OGTT. The low sensitivity for detection of abnormal glucose tolerance or diabetes with FPG testing reported is also not unexpected. In reviews on FPG or random glucose screening for undiagnosed diabetes sensitivities range from 40 to 65% [27]. Because persons tested negative on screening are not subject to subsequent confirmatory testing, the high false negative rate for FPG testing contributes to the growing number of undiagnosed cases of type 2 diabetes.

The FINDRISC score had a good sensitivity of 69% at the usually chosen 10 points cutoff level, but had poor specificity (45%). A disadvantage of several criteria in the FINDRISC and other questionnaires, like physical activity or use of fruits, is that they are often not objectively measured,and may be dependent on the availability of assistance by professionals [8]. Nevertheless, the FINDRISC has been suggested to be currently the best available diabetes screening tool for use in clinical practice in Caucasian populations [27]. Other suggested cutoff levels than the ≥10 point score (chosen for approximately equal sensitivity to the other tools) leads in our study to an even worse balance between sensitivity (for a suggested ≥14 point cutoff level falling far below that of the other tools) and the already low specificity (for a ≥7 point cutoff). Nevertheless, choosing a lower cut-off level might be considered when using a second-step confirmatory test like the FPG. In our study the specificity of this 2-step combination of FINDRISC >7 followed by FPG had a good specificity (81%), but still low sensitivity (52%).

Our study was limited in size, and performed in a selected group of persons with a moderately high risk of diabetes and IGT. However, the higher specificity of skin AF alone for IGT/diabetes detection than current detection tools, and the improvement in sensitivity without loss in specificity by conditionally adding some questions, shows the major potential of skin AF in early detection of IGT/diabetes, even in a cohort with considerable (cardiovascular) comorbidity (44%) when the specifity of skin AF is challenged. As indicated above, confirmation in larger and lower risk groups is warranted. This will also allow to define possible alternative cutoff levels of skin AF, and the selection of other characteristics included in the decision tree, depending on a priori risk. A limitation of the decision model is that information on use of antihypertensive medication, and on family history of diabetes may be unreliable. Recent decision trees with good performance for identifying individuals with IGT/diabetes have used systolic blood pressure besides age [20].

One might argue that the predictive value of FPG or HbA1c might similarly be improved by addition of questionnaire-based information (like BMI, family history), but this was not the case. Also the two-step approach with FINDRISC questionnaire first, conditionally followed by FPG, lagged far behind in performance. So, the added value of this information is higher for SAF-DM. In several previously studied non-diabetic and diabetic cohorts the relation between SAF and BMI was very modest [21], [23].This may be a factor explaining the observed additive value of BMI in IGT-dabetes risk prediction.

Skin AF and the derived decision tree may have potential for clinical implementation in diabetes prevention programs. As for monitoring effectiveness, of diabetes prevention programs, most focus on lifestyle changes including weight control. Sofar, no data is available on changes in skin AF over time in persons with IGT, and their possible value in identifying higher risk of transition to diabetes. Nevertheless, the dependence of the current decision tree on BMI subclasses does already affect to some extent its change over time. Future studies are needed tol reveal whether the potential of the current skin AF-decision tree also extends to monitoring effectiveness of diabetes prevention programs.

### Conclusion

Our skin AF based decision tree has a diagnostic performance for diabetes and IGT equal or superior to conventional risk predictors, in at least intermediate risk groups. Validation of this model in lower risk groups is still needed. The ease of use in a point of care setting, its noninvasive character, and the immediately available result qualify the skin AF based decision tree as a new tool for detection of diabetes and IGT, with a potential for clinical implementation in diabetes prevention programs. This is also supported by the previously proven additive value of skin AF in predicting complications of diabetes.
